# Two Binges of Ethanol a Day Keep the Memory Away in Adolescent Rats: Key Role for GLUN2B Subunit

**DOI:** 10.1093/ijnp/pyv087

**Published:** 2015-08-06

**Authors:** Benoit Silvestre de Ferron, Khaled-Ezaheir Bennouar, Myriam Kervern, Stéphanie Alaux-Cantin, Alexandre Robert, Kevin Rabiant, Johann Antol, Mickaël Naassila, Olivier Pierrefiche

**Affiliations:** INSERM ERI-24, GRAP, Groupe de Recherche sur l’Alcool et les Pharmacodépendances, Université Picardie Jules Verne, Bât. CURS, CHU-Sud, Amiens, France (Mr Silvestre de Ferron, Bennouar PhD, Kervern PhD, Alaux-Cantin PhD, Mr Robert, Mr Rabiant, Mr Antol, Naassila PhD, and Pierrefiche PhD).

**Keywords:** hippocampus, long-term synaptic depression, binge, ethanol, NMDA, cognition

## Abstract

**Background:**

Binge drinking is common in adolescents, but the impact of only a few binges on learning and memory appears underestimated. Many studies have tested the effects of long and intermittent ethanol exposure on long-term synaptic potentiation, and whether long-term synaptic depression is affected remains unknown.

**Methods:**

We studied the effects of one (3g/kg, i.p.; blood ethanol content of 197.5±19mg/dL) or 2 alcohol intoxications (given 9 hours apart) on adolescent rat’s memory and synaptic plasticity in hippocampus slice after different delay.

**Results:**

Animals treated with 2 ethanol intoxications 48 hours before training phase in the novel object recognition task failed during test phase. As learning is related to NMDA-dependent mechanisms, we tested ketamine and found the same effect as ethanol, whereas D-serine prevented learning deficit. In hippocampus slice, NMDA-dependent long-term synaptic depression was abolished 48 hours after ethanol or ketamine but prevented after D-serine or in a low-Mg^2+^ recording medium. Long-term synaptic depression abolition was not observed 8 days after treatment. An i.p. treatment with MK-801, tetrahydroisoxazolopyridine, or muscimol was ineffective, and long-term synaptic potentiation, intrinsic excitability, and glutamate release remained unaffected. The input/ouput curve for NMDA-fEPSPs was shifted to the left 48 hours after the binges with a stronger contribution of GluN2B subunit, leading to a leftward shift of the Bienenstock-Cooper-Munro relationship. Interestingly, there were no cellular effects after only one ethanol injection.

**Conclusion:**

Two ethanol “binges” in adolescent rats are sufficient to reversibly abolish long-term synaptic depression and to evoke cognitive deficits via a short-lasting, repeated blockade of NMDA receptors only, inducing a change in the receptor subunit composition. Furthermore, ethanol effects developed over a 48-hour period of abstinence, indicating an important role of intermittence during a repeated long-duration binge behavior.

## Introduction

Adolescence represents a critical period in terms of drugs of abuse and brain functioning. The adolescent brain is more susceptible to ethanol-induced memory impairment (White and Swartzwelder, 2005) or brain damage (Crews et al., 2000) than the adult brain. Humans experiencing alcohol during adolescence, relative to those who started drinking at the age of 21 years, have more risk to develop addiction in their life (Hingson et al., 2006). Adolescent or young adult humans drink alcohol following a pattern called “binge drinking,” defined as rapidly drinking large amounts of alcohol, and young binge drinkers have impaired visual and spatial working memory and lack control of impulsivity (Townsend and Duka, 2005; Crego et al., 2010). Binging is performed time to time, introducing some discrete periods of withdrawal from alcohol. Although such a pattern of drinking becomes common in adolescents, the impact of only a few alcohol intoxications on learning and memory appears underestimated or even neglected when considering academic performance, revealing the need for a better understanding of both the short-term and long-term effects of a few binges on cognitive function. At the preclinical level, adolescent rats are more resistant to hypnotic and ataxic alcohol-related effects (Silveri and Spear, 1998) but are more sensitive to alcohol-induced neurotoxicity and memory impairment (Markwiese et al., 1998). Many studies had submitted laboratory animals to forced binge-like exposure to determine the effects on brain neuronal networks and animal behavior. Interestingly, most of these studies use a pattern of exposure based on a “2-days-on, 2-days-off” procedure during 2 to 3 weeks (Roberto et al., 2002, 2003; Nelson et al., 2005; Pascual et al., 2007; Alaux-Cantin et al., 2013; Briones and Woods, 2013; Fleming et al., 2013). In this context, our laboratory demonstrated that such a pattern of ethanol exposure during adolescence in rats increased motivation and preference for alcohol intake later in life (Alaux-Cantin et al., 2013). However, analysis was performed some hours or many days after the end of a long intermittent treatment and hence did not determine when the cerebral modifications due to ethanol start. Thus, whether a few binges of ethanol are deleterious to cognitive function is unknown. Neither is anything known about the minimum quantity of ethanol necessary to induce cognitive deficits (Ehlers and Criado, 2010), and the role of the intermittence is not fully understood. Some of the consequences of intermittent ethanol treatment in rats do, however, concern synaptic plasticity in the hippocampus, which underlie learning and memory processes. Long-term synaptic potentiation (LTP) decreased for a few days after a 2-week intermittent ethanol exposure (Roberto et al., 2002, 2003), but whether long-term synaptic depression (LTD) is altered and which of these signals is most sensitive to ethanol remains unknown. Importantly, LTD may have a specific role in the transition to addiction to drugs of abuse in rats (Kasanetz et al., 2010) and has a role in memory (Ge et al., 2010; Dong et al., 2012, 2013). The aims of the present study were to answer 3 questions: (1) When do cognitive deficits start during a forced binge-like ethanol exposure? (2) What would be the consequences of a few binges on synaptic plasticity in the hippocampus, and do we need a high number of binges to induce any disturbances? and (3) Does the period of abstinence have any role on such disturbances? Therefore, we submitted adolescent rats to 1 or 2 binges (ie, one cycle of 2 binges) of ethanol and recorded cognitive performance and synaptic plasticity in hippocampal slices at different delay after treatment. We also investigated the mechanisms of action of ethanol using NMDA and GABA_A_ receptors related agents.

## MATERIALS AND METHODS

All experiments were performed in conformity with the European Community Guiding Principles for the Care and Use of Animals (2010/63/UE, CE Off. J. 20 October 2010), the French decree no. 2013–118 (French Republic Off. J., 2013), and the Comité Régional d’Ethique en Matière d’Expérimentation Animale de Picardie (CREMEAP). All results were obtained on male Sprague Dawley rats during late adolescent period (35–45 days) (Schneider, 2013).

### Ethanol Exposure and Pharmacological Treatments

Animals received 1 or 2 i.p. injections of ethanol (3g/kg; 20% vol/vol; Alaux-Cantin et al., 2013) administered 9 hours apart. Because learning and memory function is related to NMDA-dependent mechanisms and ethanol’s targets include both NMDA and GABA_A_ receptors, we tested i.p. administration of MK-801 (2mg/kg), ketamine (25mg/kg), D-serine (300mg/kg), tetrahydroisoxazolopyridine (THIP), or muscimol (10mg/kg each) following the same procedure as for ethanol. Dosages of drugs were chosen to specifically avoid stereotyped behavior (Watanabe et al., 2010), psychotic-related events (Manahan-Vaughan et al., 2008), or negative effect on cognition (Duffy et al., 2008; Karasawa et al., 2008; Zhang et al., 2008). Control groups included age-matched animals injected with saline.

### Blood Ethanol Content

We measured blood ethanol content (BEC) at different time points during the 2 binge-like ethanol exposures: 0.5 hours after the first injection (3g/kg, i.p.), 9 hours later just before the second injection, and the day after the second injection. Blood samples were collected from the tail vein in vials (10–20 µL) and centrifuged at 14 000 *g* for 10 minutes. The supernatant was collected and frozen at -80°C into an oxygen-rate alcohol analyzer (Analox Instruments, London) for later analysis.

### Slices and Electrophysiology

After anaesthesia with halothane and decapitation, the brain was immersed in ice-cold artificial cerebrospinal fluid (aCSF) and glued into a vibratome (Leica VT1000E, Rueil-Malmaison, France), and slices from dorsal hippocampi (400 µm thick) were cut. Selected slices were stored (>60 minutes) in an aCSF reservoir gassed with carbogen (95% O_2_/5% CO_2_; pH 7.2–7.4; 28°C) of the following composition (mM): NaCl, 125; KCl, 3; NaH_2_PO_4_, 1.25; CaCl_2_, 2.3; MgCl_2_, 1.3; NaHCO_3_, 25; glucose, 10. Hippocampus slices were transferred to a superfused recording chamber continuously perfused at a rate of 6mL/min. Synaptic response was evoked by electrical stimulation of the Schaffer collateral- commissural afferent pathway using a bipolar stimulating electrode (Phymep, Paris) at a frequency of 0.033 HZ (square pulse, duration 100 µs) for experiments on LTD and 0.1 Hz for LTP. For plasticity measurements, extracellular recordings were made with 3M NaCl (1–3 MΩ) filled glass microelectrodes into the pyramidal cell body layer of CA1, because the effects of ethanol are longer and stronger at the soma level (Roberto et al., 2002). An input/output relationship was performed to determine the response parameters for each slice. Stimulus strength was increased in 20-µA steps until the maximal response amplitude and intensity of the test pulses was set to 50%-60% of the maximal amplitude. Signals were amplified (Grass amplifier, x1000-2000), filtered (1–3 KHz), and acquired on computer with Signal software (CED, Cambridge, UK) for off-line analysis. Responses were quantified by measuring population spike amplitude. LTD was induced with a train of 1-Hz frequency stimulation made of a pair of pulses separated by 200ms and delivered 900 times (pLFS200-900) (Kemp et al., 2000). LTP was induced with 3 stimulations of 1-second duration delivered at 100 Hz, separated by 10 seconds. Paired-pulse facilitation, tested during baseline recording, consisted in double pulse stimulation at test intensity with an interpulse interval of 50-100-150-200ms. The ratio between the second over the first pulse gave the percent of facilitation. For field excitatory postsynaptic potentials (fEPSPs) analysis, the recording electrode was placed into the *stratum radiatum*. NMDA-fEPSPs were recorded in a zero-Mg^2+^ aCSF containing 50 µM bicuculline and 10 µM 6-cyano-7-nitroquinoxaline-2,3-dione, an AMPA/kainate receptor antagonist. At the end of recording, 20 µM DL-2-amino-5-phosphonopentanoic acid (AP-5) was added to check for the NMDA dependency of the EPSP. The EPSP’s slope was measured after the afferent volley and before the full amplitude of the EPSP. Results are expressed as percent change to baseline value. The role of GluN2B subunit was assessed using 5 µM Ro 25–6981, a selective antagonist of GluN2B subunit (Fischer et al., 1997). Only slices demonstrating 10 minutes of stable baseline recording were used, and measurements were averaged every minute. Recordings were performed 24 or 48 hours after treatment to unravel the influence of intermittence during a binge-like exposure. For the recovery of ethanol’s effects, this delay was increase to 8 days. For electrophysiology, n is the number of slices tested. A maximum of 3 slices/animal was used.

### Novel Object Recognition Test

**Figure 1.**
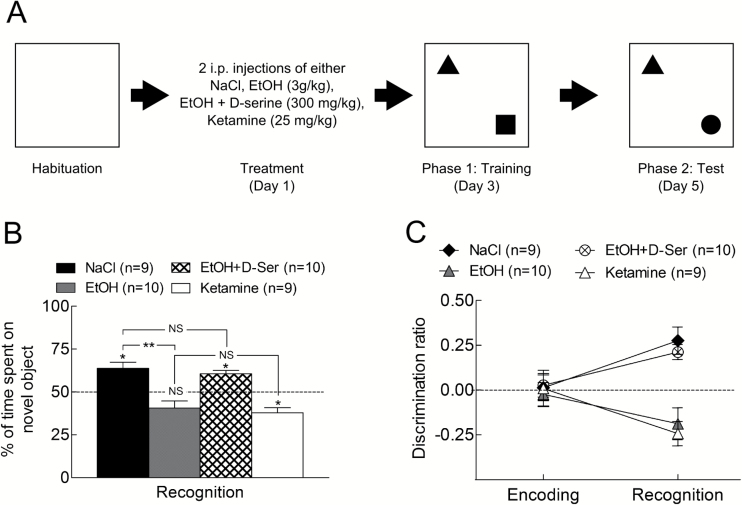
Performances in novel object recognition (NOR) task. (A) Time line for the NOR test. (B) Percent of time spent on the novel object during recognition phase after different pharmacological treatments. After 2 binges of ethanol (3g/kg, i.p.), animals spent less time on the novel object compared with the saline group. Administration of D-serine (300mg/kg, i.p.) in addition to EtOH prevented the low performance induced by EtOH alone. Administration of ketamine (25mg/kg, i.p.) induces the same result as EtOH. (C) Discrimination ratio for the groups presented in A. During recognition phase, animals treated with either NaCl or EtOH+D-serine displayed a high ratio, revealing their preference for the novel object. In contrast, EtOH or ketamine treatments induced a lower ratio, suggesting there was no preference for the novel object.

Thirty-day-old animals (n = 45) were allowed to acclimate to the facility and handled twice a day. One week prior to the experiment, they were i.p. injected with saline once per day and introduced into the test box at different times to restrict novelty to the objects and reduce anxiety. Two rats, not tested for behavior, were placed into the test box for 15 minutes every day to eliminate extraneous olfactory stimuli. Rats were transferred to the test room 30 minutes before the experiment. We used a square box with an open top, made of opaque Plexiglas (45×45×45cm) with a 30-lux illumination. The box was furnished with bedding and cleaned between sessions. The objects, also cleaned between sessions, were chosen to avoid any preference but were different enough in shape to be distinguished. They were too heavy to be displaced by rats and were located at the same distance from the wall. Presentation of the objects was counterbalanced: one-half of the rats were presented object B as novel, whereas the other one-half were presented object C as novel. After a 10-minute habituation, animals were submitted to a 10-minute training phase (phase 1, Figure 1A) with 2 objects in a diagonal configuration. Phase 2 (test) was performed 48 hours later by replacing one object. The delay allows comparison with studies using 2 days off during intermittent exposure (Pascual et al., 2007, 2009; Alaux-Cantin et al., 2013). Digital video acquisition system (Pinnacle Studio HD v.15 software) was used. Off-line analysis consisted in measuring the total exploring time spent on the objects during the first 5 minutes of recording. We considered all behaviors as sniffing, licking, or touching the objects with forelimbs. Exploration time was normalized and expressed as a percentage of time spent by a rat on novel object compared with the familiar object. Rats displaying low motor activity or low explorative (<10 seconds for both objects) behaviors were excluded. This procedure is a one learning trial, one test trial procedure, allowing assessment of an acute pharmacological treatment on a single learning experience. Novel object preference (discrimination ratio) was quantified using novel − familiar)/(novel + familiar) calculation. Scores close to zero reflect no preference, positive values reflect preference for the novel object, and negative values reflect preference for the familiar object.

We evaluate the effect of drugs on the training phase. Thus, rats were treated 48 hours before training (Figure 1A) with 2 i.p. injections separated by a 9-hour interval of one of the following: NaCl 0.09%, EtOH 3g/kg, EtOH 3g/kg + D-serine 300mg/kg, ketamine 25mg/kg. Each group included 8/10 animals.

### Statistics and Data Presentation

All data are expressed as mean±SEM. Statistical analysis of synaptic plasticity was performed on raw data with unpaired Student’s *t* test. In all tests, we chose *P*<.05 as significant. In figures with electrophysiological results, traces on top are averaged and superimposed raw signal taken before and after synaptic plasticity in different conditions with time calibration of 10 ms and voltage calibration of 1 mV. Repeated-measure ANOVA (RM-ANOVA) analysis was performed for input/output curves, paired-pulse facilitation, and behavior experiments.

## RESULTS

### Blood Ethanol Content

Thirty minutes after EtOH injection (3g/kg), BEC reached 197.5±19mg/dL, and 9 hours later, before a second EtOH injection, BEC was 7.9±1mg/dL. A similar level of BEC was found (7.3±0.5mg/dL) the day after the second injection.

### Disturbances of Cognitive Function

We presented 2 objects 48 hours after ethanol and tested for retention in presence of a new object again 48 hours later (Figure 1A). RM-ANOVA indicated an effect of treatments [F_(4,91)_=7.8, *P*<.001], no time effect [F_(4, 91)_ = 2.1, *P*=.1] and time × treatment interaction [F_(4,91)_=6.6, *P*<.001]. Indeed, the control group treated with saline spent significantly more time on the novel object (*P*=.01; Figure 1B), whereas those receiving 2 binges of ethanol spent less time on the new object compared with control group (*P*=.003). Animals receiving a combination of EtOH+D-serine (300mg/kg i.p.) spent significantly more time on the novel object compared with the EtOH group (*P*=.001), reaching a value close to control group (*P*=.9). We next tested ketamine (25mg/kg i.p.), a short-lasting antagonist of NMDA receptors, and found that treated animals did not spend enough time on the novel object (*P *=.01). This effect was similar to EtOH (*P*=.5). Discrimination ratio (Figure 1C) shows that all groups correctly explored the 2 first objects, spending about 50% of their total exploratory time on each object. However, during the test phase, EtOH and ketamine animals failed to recognize the new object, whereas the presence of D-serine readjusted the ratio to control.

### Bidirectional Synaptic Plasticity After Two Binges of Ethanol

**Figure 2.**
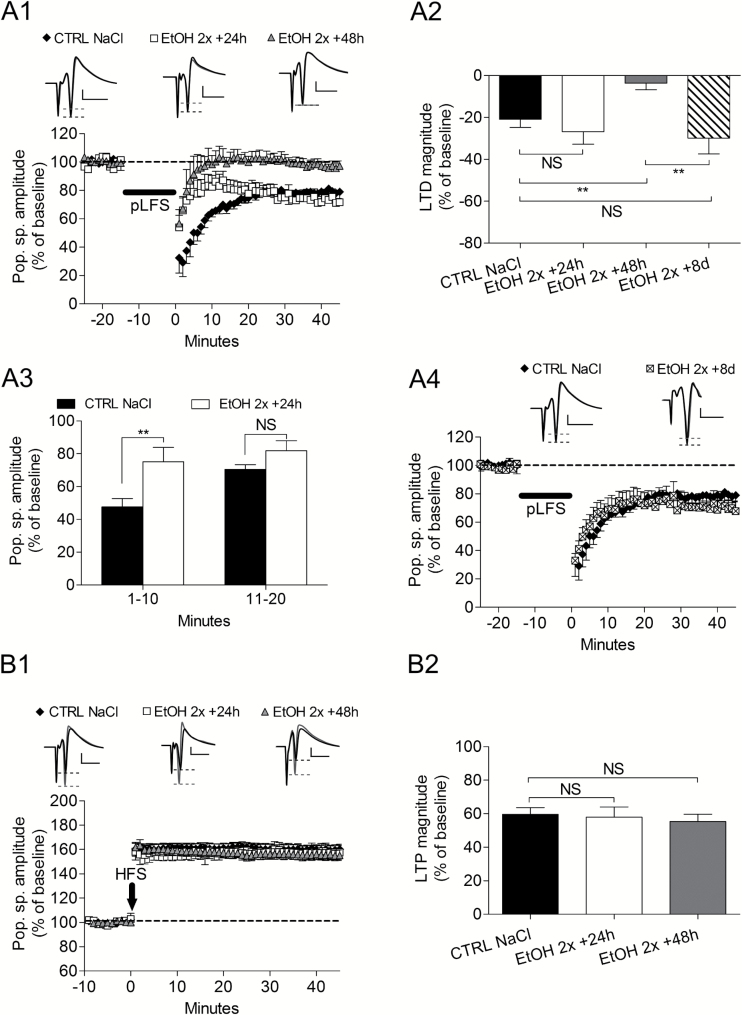
Effects of 2 i.p. injections of ethanol (3g/kg) in 1 day on bidirectional synaptic plasticity after different delays. (A1) After 2 binges of ethanol, long-term synaptic depression (LTD) magnitude at the end of recording period was abolished after a 48-hour delay. (A2) LTD magnitude as measured in different conditions. At 24h delay LTD was not changed but was abolished at a 48-hour delay. LTD abolition was transient, since at 8 days delay, LTD magnitude was similar to control. (A3) Measurements performed during the first 20 minutes following the electric stimulation inducing LTD show that signal magnitude was higher than control 24 hours after 2 binges. (A4) Eight days after 2 binges of EtOH, LTD magnitude was the same as in control slices. (B1) Long-term synaptic potentiation (LTP) was not affected by 2 binges of ethanol at any delay. (B2) Bar graphs summarizing the effects of different experimental conditions on LTP magnitude. **P<.01 and NS is for nonsignificant.

To understand the cellular mechanisms of the cognitive deficits observed after only 2 binges of ethanol, we recorded LTP and LTD on hippocampus slices of rats treated with 2 ethanol injections 24 or 48 hours after treatment. For all electrophysiological experiments, NaCl administration did not affect LTD magnitude at any delay. Twenty-four hours after EtOH, LTD magnitude at the end of the recording was not different from control (control NaCl: -20.9±3.9 % of baseline, n = 7, vs EtOH24h: -26.7±7.9 %, n = 6, *P* > 0.05; Figure 2A 1-A2). However, during the first 10min following stimulation, the evoked response was larger in the EtOH group (Control NaCl: 47.6±5.5 % of baseline, n = 7, vs EtOH1-10: 75.2±8.6 %, n = 6, *P*=.02, Figure 2A 1-A3). When recordings were performed 48h after EtOH, LTD was abolished at the end of the recording (EtOH48h: -3.6±3.2 %, n = 8, *P*=.002 compared with control) (Figure 2A 1-A2). LTD abolition was not obtained if the delay between treatment and recordings was increased to 8 days (control NaCl: -20.9±3.9% of baseline, n = 7, vs EtOH8d: -29.9±7.5, n = 6, *P*=.1) (Figure 2A 2-A4). In contrast to LTD, both magnitude and time course of LTP were not affected by ethanol at either delay (control NaCl: 59.6±4.0% of baseline, n = 8, vs EtOH 24 hours: 58.0±6.0, n = 5, *P*=.4; vs EtOH 48 hours: 55.4±4.3, n = 8, *P*=.2) (Figure 2B 1-B2). These results obtained after a 48-hour delay suggest that abolition of LTD might have been responsible for the failure in learning. To check this assumption, we recorded hippocampus slices the day after behavior experiments in 2 animals that failed in the novel object recognition (NOR) test and found that LTD was absent (data not shown). Conversely, one animal that received ethanol + D-serine and thus presented a rescued learning recorded 24 hours after the end of behavior experiment showed an LTD (data not shown).

### The Effects of One Binge of Ethanol on Synaptic Plasticity

**Figure 3.**
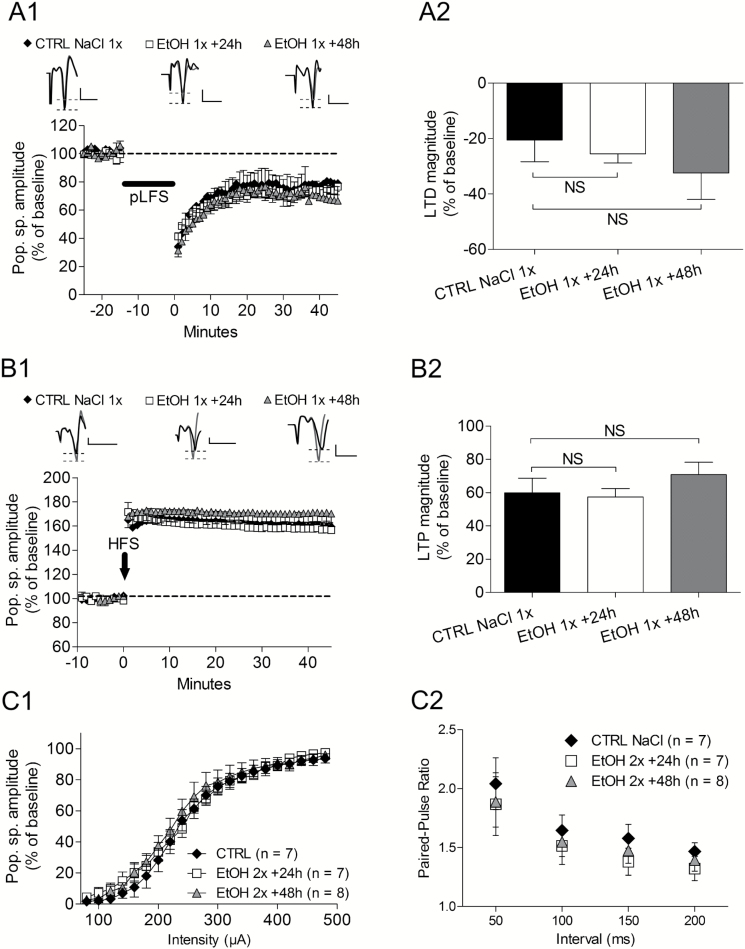
Effects of 1 i.p. injection of ethanol (3g/kg) in 1 day on bidirectional synaptic plasticity and intrinsic properties. (A1) Long-term synaptic depression (LTD) was not changed at any delay after treatment as well as long-term synaptic potentiation (LTP) (A2). (B1) Bar graph of the magnitude of LTD showing no significant changes at any delay after ethanol treatment. (B2) Bar graph illustrating the magnitude of LTP and the nonsignificant differences compared with control slice population. (C1) Superimposed input/output curves determined before conditioning stimulations in different experimental conditions (ethanol affecting synaptic plasticity or not) shows that ethanol does not change the intrinsic excitability of the network. (C2) In similar experimental conditions as in C1, paired-pulses facilitation was also not affected by ethanol.

For 24- or 48-hour time delays, the LTD magnitude was not changed by one binge of ethanol (control NaCl: -20.5±7.8% of baseline, n = 7 vs EtOH 24 hours: -25.5±3.3, n = 8, *P*=.3; vs EtOH 48 hours: -32.4±9.5, n = 4, *P*=.2) (Figure 3A 1-A2). Similarly to LTD, LTP was not affected at any delay (control NaCl: 60.0±8.8% of baseline, n = 7 vs EtOH 24 hours: 57.4±5.0, n=7, *P*=.4; vs EtOH 48 hours: 70.8±7.4, n = 4, *P*=.2) (Figure 3B 1-B2).

### Effects of Ethanol on Basal Synaptic Transmission

To identify the mechanism of ethanol inhibitory effects on hippocampus LTD, we first analyzed whether ethanol treatment affected intrinsic excitability of the hippocampus network and/or neurotransmitter release. Input/ouput relationships (Figure 3C1) were compared in animals that received saline and were recorded 48 hours later and in those receiving 2 ethanol injections and recorded 24 and 48 hours later. RM-ANOVA revealed no treatment effect [F_(2, 459)_=.27, *P*=.7], an effect of intensity [F_(2, 459)_=319.6, *P*<.001], and no interaction [F_(2, 459)_=.6, *P*=.9]. In the same populations, paired-pulses facilitation (Figure 3C2) shows no difference at any intervals. RM-ANOVA showed no treatment effect [F_(2,85)_=0.3, *P*=.7], an effect of intervals [F_(2,85)_=34.1, *P*<.001] and no interaction [F_(2, 85)_=0.2, *P*=.9].

### Cellular Targets of 2 Binges of Ethanol During LTD Abolition

**Figure 4.**
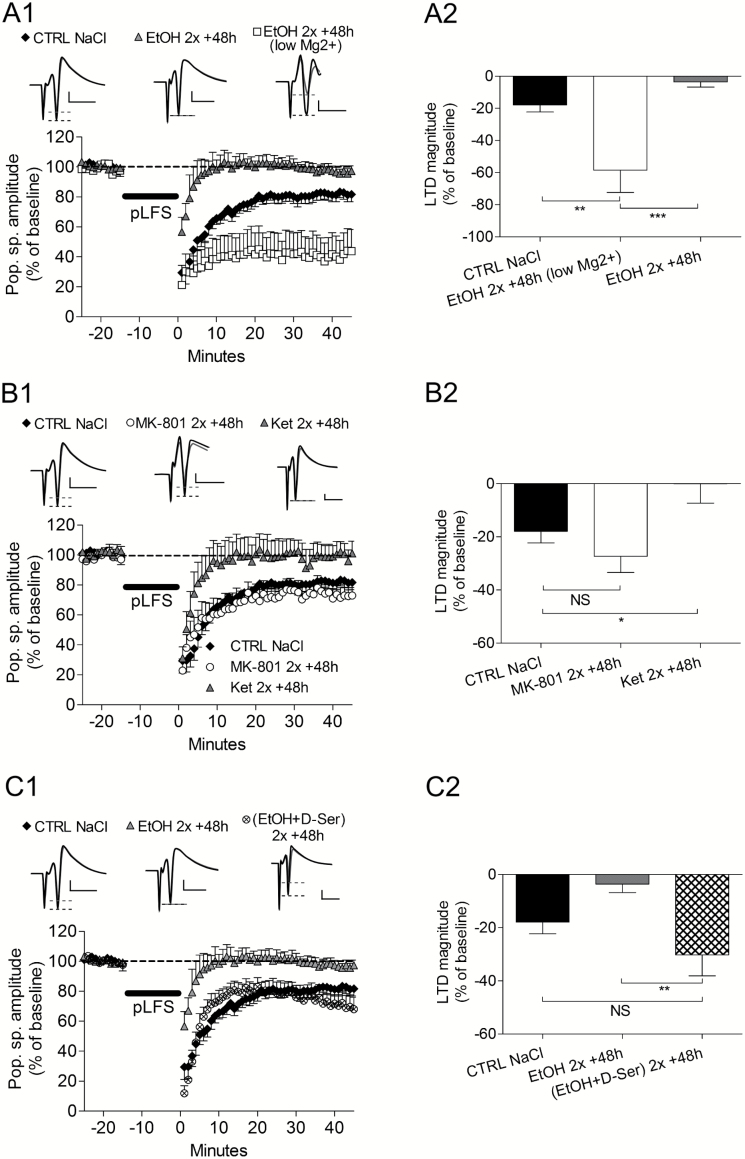
Involvement of NMDA receptors in the effect of ethanol on long-term synaptic depression (LTD) abolition. (A1) LTD was not abolished when recordings were performed 48 hours after 2 binges of ethanol in presence of a low-Mg^2+^ containing artificial cerebrospinal fluid (aCSF). (A2) Illustration of LTD magnitude in the different conditions tested. (B1) When ethanol was replaced by MK-801 i.p. injections (2mg/kg), LTD was not abolished after the 48-hour delay. In contrast, ethanol’s effects were replicated with ketamine i.p. injections (25mg/kg). (B2) Bar graph of final LTD magnitude in the different conditions tested. (C1) Coadministrations of D-serine (300mg/kg) with EtOH (3g/kg) prevented the effects of EtOH on LTD. (C2) The bar graph shows that the presence of D-serine maintained LTD magnitude at control level.

Behavior experiments suggested that NMDA-dependent processes were involved in the effect of ethanol. Using 50 µM AP-5, an antagonist of NMDA receptors, we first checked that the stimulation protocols used were inducing NMDA-dependent LTP and LTD and whether binges of ethanol changed this property. Our result showed that both LTP and LTD were AP-5 sensitive before alcohol (LTP NaCl, n=8, *P*=.4; LTD NaCl, n=8, *P*=.5), and LTP remained NMDA-dependent 48 hours after the binges (LTP EtOH, n=5, *P*=.2) (Figure 6A). To analyze further the role of NMDA receptors, we recorded slices of ethanol-treated animals (2 i.p. injections, recorded 48 hours later) in a low-Mg^2+^ aCSF allowing for the release of the voltage-dependent blockade of NMDA receptors by Mg^2+^. Here, LTD was not abolished and was even stronger (Figure 4A 1-A2, control NaCl: -18.0±4.3% of baseline, n=9 vs EtOH 48 hours low-Mg^2+^: -58.5±13.9, n=5, *P*=.002, and EtOH 48 hours low-Mg^2+^ vs EtOH 48 hours: -3.6±3.1, n=8, *P*=.0003). Based on our behavior results, we recorded hippocampal slices 48 hours after an in vivo treatment with MK-801 or ketamine, a long- and a short-lasting blocker of NMDA receptors, respectively. After MK-801, LTD magnitude was similar to control group (Figure 4B 1-B2; MK-801–48 hours: -27.3±6.2, n=6, *P*=.1 vs control group). In contrast, ketamine abolished LTD and evoked similar effects as 2 binges of EtOH (Figure 7B1-B2; Ket 48 hours: -0.03±7.0, n = 6, *P*=.02 vs control group). Interestingly, D-serine administered after each binge of EtOH prevented LTD abolition 48 hours later (Figure 4C 1-C2) (control NaCl: -18.0±4.3% of baseline, n=9 vs EtOH+D-serine 48 hours: -30.2±8.0, n = 5, *P*=.08). LTD in presence of D-serine was also significantly different from EtOH alone (EtOH 48 hours: -3.6±3.2%, n=8, vs EtOH+D-serine 48 hours: -30.2±8.0, n = 5, *P*=.002).

**Figure 5.**
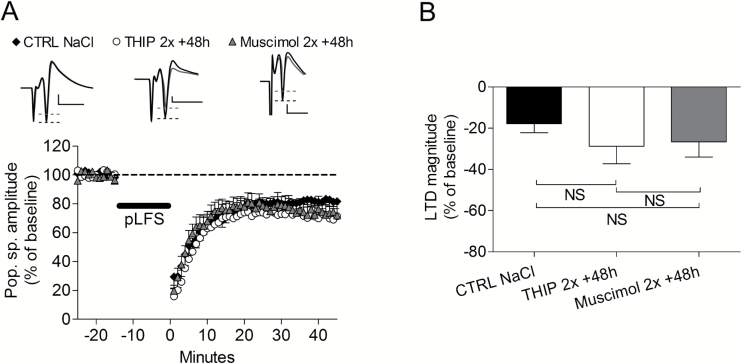
No role for GABA_A_ receptors in the effects of ethanol. (A1) Two i.p. administrations of either tetrahydroisoxazolopyridine (THIP) (10mg/kg) or muscimol (10mg/kg) instead of ethanol did not induce long-term synaptic depression (LTD) abolition after a 48-hour delay. (A2) Summary of the effects of GABA_A_ receptor agonists on LTD magnitude.

Besides NMDA receptors, GABA_A_ receptors are potentiated by ethanol. To differentiate the effects of ethanol between NMDA and GABA_A_ receptors, we used THIP and muscimol as 2 GABA_A_ receptor agonists (Figure 5A-B). None of these agents abolished LTD after 48 hours (Figure 8A-B; THIP 48 hours: -28.8±8.5, n=6 vs Musci 48 hours: -26.6±7.3, n = 6, *P*=.4). Indeed, both drugs induced similar LTD as control slices (control NaCl: -18.0±4.3, n = 9, with *P*=.1 compared with either THIP 48 hours or Musci 48 hours).

### Analysis of NMDA-fEPSPs After 2 Binges of Ethanol

**Figure 6.**
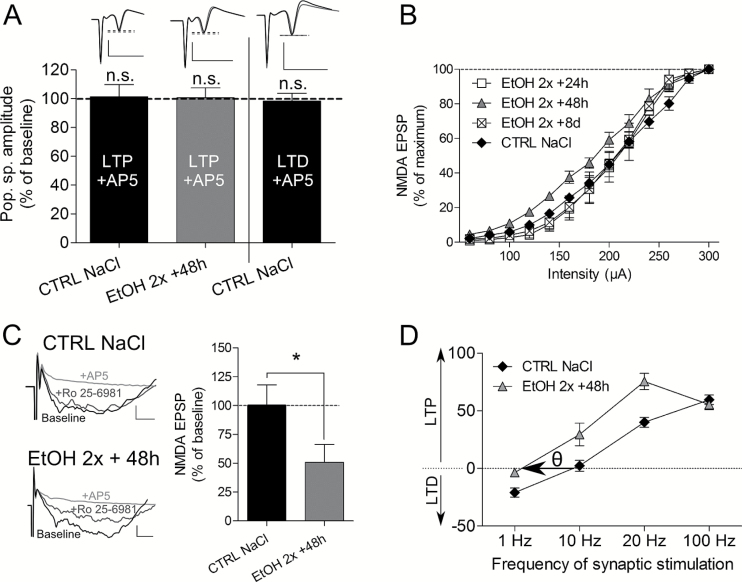
A role for GluN2B subunit in the effect of ethanol. (A) Quantification of long-term synaptic potentiation (LTP) and long-term synaptic depression (LTD) in presence of AP-5. Both signals are AP-5 sensitive and binges of ethanol do not change this property. (B) Input/output (I/O) curves performed for NMDA-fEPSPs at different time delay after the 2 binges. Only the curve at the 48-hour delay was significantly shifted to the left. (C) Effect of Ro 25–6981 on NMDA-fEPSPs in control group (NaCl) and 48 hours after 2 binges, showing that the antagonist of GluN2B subunit was effective only in EtOH slices. Calibration bars are 10ms for time and 0.05 mV for amplitude. (D) Relationship between magnitude of plasticity and stimulation frequency in control slices (NaCl) and 48 hours after EtOH (EtOH 2×+48 hours) showing a leftward shift of the modification threshold (*θ*) for synaptic plasticity after EtOH.

One possibility to explain LTD abolition would be to consider an inhibition of NMDA-dependent EPSP 48 hours after 2 binges. We thus performed input/output (I/O) curves of NMDA-fEPSPs at 24-hour, 48-hour, and 8-day delays after the binges. The results showed that I/O curves at both 24-hour and 8-day delays were not significantly different from the control group (NaCl, n = 7 vs EtOH 8 days, n = 3, *P*=.9; NaCl, n = 7 vs EtOH 24 hours, n = 4, *P*=.8) (Figure 6B). However, the I/O curve established at the 48-hour delay (n = 7) was displaced to the left compared with the NaCl group (*P*=.04). This result show that inducing an NMDA-fEPSP is facilitated at a 48-hour delay and that this change is transient. To better understand ethanol-induced changes in NMDA-fEPSPs, we hypothesized that ethanol modified the subunit composition of the NMDA receptor and possibly increased GluN2B subunit. We then assessed the role of the GluN2B subunit using 5 µM Ro 25–6981, a selective antagonist of GluN2B. We found that in control slices, Ro 25–6981 had no effect on NMDA-fEPSPs, whereas 48 hours after 2 binges, fEPSP magnitude was decrease by 50% (EtOH 48 hours, n = 6 vs control, CTRL n = 6, *P*=.04) (Figure 6C).

### Two Binges of Ethanol Change the Threshold for Synaptic Plasticity

Our data suggested that EtOH may have affected the threshold to induce either LTP or LTD at a 48-hour delay. To prove this assumption, we established a Bienenstock-Cooper-Munro (BCM) curve (Bienenstock et al., 1982) by testing different stimulation frequencies while recording plasticity (Figure 6D). With 2×10 Hz stimulation no LTP was induced in control slices (n = 4), whereas an LTP was obtained in EtOH slices (n = 2). Increasing stimulation to 2×20 Hz increased the LTP magnitude in the 2 populations with a higher level in EtOH slices. However, 3×100 Hz led to a comparable LTP magnitude (CTRL, n = 8; EtOH, n = 8), while a 1-Hz stimulation led to an LTD in control slices (n = 7) but no plasticity in EtOH slices (n = 8). Therefore, 2 binges of ethanol lead to a leftward shift of the BCM curve.

## Discussion

We studied the impact of a few alcohol binges on learning and on hippocampal mechanisms underlying learning and memory in adolescent rats. Our major findings are: (1) cognitive deficits are induced 48 hours after only 2 alcohol binges; (2) ethanol-induced abolition of LTD in the hippocampus is observed at the same time when cognitive deficits occurred; (3) both cognitive and LTD deficits are prevented by D-serine and mimicked by ketamine; and (4) deficits seem to be linked to repetitive short-lasting NMDA receptor blockade only, inducing a change in subunit composition of the NMDA receptor. These results are of particular relevance, since the impact of a few alcohol intoxications is often underestimated and/or neglected, particularly by underage people.

### Behavior Effects of Two Binges of Ethanol

Two binges of ethanol induced cognitive deficits, since NOR was impaired. More precisely, animals treated with ethanol failed to recognize a novel object during test phase, probably because of a deficit during the acquisition phase. Deficit does not concern exploratory behavior, since ethanol-treated animals spent 50% of the time on the first 2 objects similarly to control group. Interestingly, the delay between the 2 ethanol binges and behavior training in NOR was beyond the time necessary to eliminate ethanol (ie, 48 hours) but still effective in disturbing the test phase. More interestingly, animals were trained at a time when hippocampus LTD has been selectively suppressed by ethanol (see below). There are many studies indicating that LTD is important for recognition memory. Novelty acquisition required LTD in the hippocampus (Ge et al., 2010; Dong et al., 2012, 2013) or facilitates its induction (Manahan-Vaughan and Braunewell, 1999; Kemp and Manahan-Vaughan, 2004), and object recognition is disturbed after viral transfection, which selectively abolished LTD (Griffiths et al., 2008). LTD was absent the day after the end of behavior experiments (ie, 5 days after ethanol treatment), further suggesting that lack of LTD was involved in learning deficit. Interestingly, this 5-day delay corresponds to a time point where LTP into the hippocampus had recovered from a 2-week intermittent ethanol exposure with a BEC of 180mg/dL (Roberto et al., 2002, 2003). Unfortunately, there is a paucity of data concerning short-lasting systemic ethanol exposure and learning. In 7-day-old animals, 2 i.p. doses of 2.5g/kg ethanol given 2 hours apart impaired Y-maze performance (spontaneous alternation) without affecting water maze learning and retrieval 3 weeks later (Izumi et al., 2005a), although others found some disturbances (Wozniak et al., 2004). Four days of binge ethanol exposure in young adult rats impaired NOR 1 week after treatment (Cippitelli et al., 2010), suggesting that repetition of binges durably alter cognitive function. However, in this study, the dose of ethanol was higher than in the present study (9–15g/kg/d) and no electrophysiological experiments were performed. Furthermore, 15 days after a chronic methylphenidate treatment but not the day after the treatment (Taukulis et al., 2014), object memory was impaired, suggesting that the effects of drugs of abuse develop during the abstinence period. Considering alcohol had been totally eliminated at the time of the training phase, our results reveal that ethanol-induced disturbances last much longer than the time necessary for alcohol elimination.

### LTD Is More Sensitive Than LTP

Our study is to our knowledge the first one investigating concomitantly bidirectional synaptic plasticity in rat hippocampus slices and cognition after a short-duration, binge-like ethanol exposure. Similarly to behavior experiments, there is a lack of data regarding synaptic plasticity after very short-lasting systemic ethanol exposure. Interestingly, one dose of ethanol did not affect synaptic plasticity in rats, whereas 2 binges of ethanol were effective, suggesting that this is a threshold value for ethanol in rats. To our knowledge, this is the first time that a threshold can be suggested in the effects of ethanol at both cellular and behavior levels. A striking result was that LTD was abolished whereas LTP was not altered, revealing a possible higher sensitivity of LTD towards EtOH effects. This remains surprising, because the stimulations used elicit NMDA-dependent synaptic plasticity at this age (Kemp et al., 2000; Liu et al., 2004), meaning we should have observed a decrease of LTP as well. One possibility to explain this result is that mechanisms of LTP reduction by ethanol are more complex than simply an inhibition of the NMDA receptors as shown for in vitro ethanol application (Izumi et al., 2005b). Another possibility would be to consider that LTP has been reduced previously to LTD and that the neuronal network compensated for this reduction after 48 hours. Indeed, one study reported an elevation of BDNF mRNA in the hippocampus (Kulkarny et al., 2011) with a BEC of 174mg/dL 2 hours after 2-hour ethanol vapours. Therefore, a role for BDNF may be envisaged in our experiments. Another solution would be to consider a ceiling effect for LTP due to the high stimulation used (see below). The sensitivity of LTD together with a lack of effect on LTP was probably due to the low level of BEC we obtained and is probably not related to brain damages (Collins et al., 1996). Nevertheless, abolition of LTD have been described up to 3 weeks after 2 doses of 2.5g/kg ethanol given i.p. 2 hours apart at postnatal day 7 (Izumi et al., 2005a). In adult rats, chronic intermittent ethanol exposure reduced hippocampus NMDA-dependent LTD (Thinschmidt et al., 2003), although ethanol treatment was performed over several weeks. Another assumption is that sensitivity of LTD does not rely only on the function of NMDA receptors but possibly involved the intracellular signalling cascade leading to LTD.

### The Effects of Ethanol Include a Specific Role for GluN2B Subunit

At the behavior level, learning deficit was absent when D-serine, a co-agonist of the NMDA receptor, was co-injected with ethanol. D-serine is a pro-mnesiant drug and acute ethanol is known to block NMDA receptors (Möykkynen and Korpi, 2012). Thus, our results suggest that D-serine overcame or prevented the inhibition of NMDA receptors produced by each dose of ethanol. Furthermore, ethanol-induced LTD abolition was prevented when D-serine was i.p. co-injected with ethanol. Cognitive impairment was mimicked with ketamine, a short-lasting antagonist of NMDA receptors, while at cellular level LTD abolition was also obtained with ketamine. Altogether, these results support the hypothesis that binges of ethanol inhibit NMDA receptors, inducing LTD abolition and leading to learning deficit 48 hours after EtOH. This suggestion is supported by animals co-injected with EtOH and D-serine, which showed an LTD the day after the end of the behavior experiments, further revealing the concomitancy of correct learning in presence of an NMDA-dependent LTD in the hippocampus. Collectively, both behavior experiments and electrophysiological data strongly support the assumption that LTD is important for such learning tasks. This makes the present study the first one to suggest an association between NOR, binge-like ethanol exposure, and LTD. Interestingly, i.p. injections of MK-801, a long-lasting NMDA receptor antagonist, did not mimic the effects of ethanol at the cellular level, suggesting that blockade of NMDA receptors by ethanol should be of short duration. Additionally, the fact that 1 binge was ineffective, in contrast with 2 binges, further suggests that NMDA receptors blockade should be repeated in order to induce disturbances at the cellular level. Finally, and in contrast to NMDA receptor involvement, a role for a GABAergic component can be excluded, since no LTD inhibition was observed after i.p. injections of 2 different agonists of the GABA_A_ receptor.

To further elucidate the mechanisms of EtOH effects on bidirectional NMDA-dependent plasticity, we checked whether NMDA-fEPSP were disturbed at different delays after the binges. I/O curves for NMDA-fEPSPs showed that they were present at all time points and that, importantly, only the curve at the 48-hour delay was shifted leftward, revealing that abolition of NMDA-dependent LTD was concomitant to a stronger NMDA-fEPSPs and that such an increase was transient. Interestingly, at the 48-hour delay, the higher NMDA-fEPSP was accompanied with a stronger effect of a specific GluN2B subunit antagonist compared with control slices. Additionally, establishing the BCM computational model of synaptic plasticity (Bienenstock et al., 1982), 2 binges of ethanol shifted the modification threshold for LTP and LTD to the left at the 48-hour delay. Such a shift has been related to changes in the GluN2A to GluN2B ratio. Specifically, when the ratio is low (ie, when GluN2B>GluN2A), LTP is more likely to occur rather than LTD, whereas when the GluN2A to GluN2B ratio is high, LTD is favored (for review, see Paoletti et al., 2013). Taken together, our results argue in favor of a low GluN2B to GluN2A ratio and thus a possible increase in GluN2B subunit expression. We then may suggest that LTD abolition was due to an upregulation of GluN2B subunit, leading to a leftward shift of the synaptic threshold. Further, the lack of effect of EtOH on NMDA-dependent LTP at the 48-hour delay may derive from the high-frequency stimulation used, which was possibly too strong. This would explain why we did not observe a higher LTP as previously described when GluN2B subunits were upregulated (for review, see Shipton and Paulsen, 2013). Accordingly, an increase in GluN2B subunit after binges of EtOH may induce a compensatory mechanism, maintaining LTP at control level.

### Intermittence Reveals the Effects of Ethanol

We varied the delay between treatments and recordings, submitting the rat to a relatively short (24 hours) or long abstinent period (48 hours) such as those that may appear in adolescent humans. Thus, an interesting result is the fact that ethanol-induced disturbances appear gradually over 48 hours following the treatment. We suggest this delay may be necessary to upregulate the GluN2B subunit. In this context, it is interesting to note that most of the studies about binge-like ethanol exposure include 2 days off between each exposure. Furthermore and as discussed above, the repetition of ethanol injections is necessary to alter synaptic plasticity.

### Significance of BEC Level

French reports concerning adolescents’ hospital admission for acute ethanol intoxication reported a BEC of 150 to 170mg/dL (CIRDD, 2011 and AIRDDS-CIRDD, 2013), which is close to what we measured (aproximately 200mg/dL). There was no ethanol in the blood before the second injection, mimicking the recovery period between successive binges in humans. Therefore, our procedure can be interpreted as 2 independent intoxications inducing a repeated specific ethanol-related mechanism, leading to behavior and electrophysiological disturbances. In several studies, ethanol regimen induced 500mg/dL BEC 1 hour after a second injection and/or a sustained 200-mg/dL level for several hours (Collins et al., 1998; Crews et al., 2000; Izumi et al., 2005a). With these regimen, animals showed neurodegeneration in the hippocampus (Collins et al., 1998; Crews et al., 2000) but not when BEC was <300mg/dL (Collins et al., 1996). In comparison, brain damage, if present, was set at a minimum in our conditions.

In summary, 48 hours after only 2 binges of ethanol in adolescent rats, LTD was transiently abolished for several days. LTD abolition needs 48 hours to be measurable and was responsible for learning deficits. We suggest that LTD abolition occurred through a repeated short-lasting blockade of NMDA receptors only, inducing an upregulation of GluN2B subunit and leading to a shift in the threshold level for synaptic plasticity. Because only a few binges of ethanol have the potential to induce deleterious cellular and behavior effects in relation to cognitive functions, we suggest that binge-drinking behavior during adolescence should be highly considered right at the beginning of such a pattern of consumption.

## Statement of Interest

None.

This work was supported by IREB, MildT, the European Program INTERREG IVA–Alcobinge, the Conseil Régional de Picardie, and The European Foundation for Alcohol Research (EA 12 28).

## References

[CIT0001] Alaux-Cantin S Warnault V Legastelois R Botia B Pierrefiche O Vilpoux C Naassila M (2013) Alcohol intoxications during adolescence increase motivation for alcohol in adult rats and induce neuroadaptations in the nucleus accumbens. Neuropharmacology 67:521–331. 23287538 10.1016/j.neuropharm.2012.12.007

[CIT0002] Briones TL Woods J (2013) Chronic binge-like alcohol consumption in adolescence causes depression-like symptoms possibly mediated by the effects of BDNF on neurogenesis. Neuroscience 254:324–334. 24076087 10.1016/j.neuroscience.2013.09.031 PMC3983287

[CIT0003] Cippitelli A Zook M Bell L Damadzic R Eskay RL Schwandt M Heilig M (2010) Reversibility of object recognition but not spatial memory impairment following binge–like alcohol exposure in rats Neurobiol Learn Mem 94:538–546. 20849966 10.1016/j.nlm.2010.09.006 PMC2975859

[CIT0004] Collins MA Corso TD Neafsey EJ (1996) Neuronal degeneration in rat cerebrocortical and olfactory regions during subchronic “binge” intoxication with ethanol: possible explanation for olfactory deficits in alcoholics. Alcohol Clin Exp Res 20: 284–292. 8730219 10.1111/j.1530-0277.1996.tb01641.x

[CIT0005] Collins MA Zou JY Neafsey EJ (1998) Brain damage due to episodic alcohol exposure in vivo and in vitro: furosemide neuroprotection implicates edema-based mechanism. FASEB J 12:221–230. 9472987 10.1096/fasebj.12.2.221

[CIT0006] Crego A Rodriguez-Holguín S Parada M Mota N Corral M Cadaveira F (2010) Reduced anterior prefrontal cortex activation in young binge drinkers during a visual working memory task. Drug Alcohol Depend 109:45–56. 20079980 10.1016/j.drugalcdep.2009.11.020

[CIT0007] Crews FT Braun CJ Hoplight B Switzer RC 3rd Knapp DJ (2000) Binge ethanol consumption causes differential brain damage in young adolescent rats compared with adult rats. Alcohol Clin Exp Res 24:1712–1723. 11104119

[CIT0008] Dong Z Gong B Li H Bai Y Wu X Huang Y He W Li T Wang YT (2012) Mechanisms of hippocampal long-term depression are required for memory enhancement by novelty exploration. J Neurosci 32:11980–11990. 22933783 10.1523/JNEUROSCI.0984-12.2012 PMC3774153

[CIT0009] Dong Z Bai Y Wu X Li H Gong B Howland JG Huang Y He W Li T Wang YT (2013) Hippocampal long-term depression mediates spatial reversal learning in the Morris water maze. Neuropharmacology 64:65–73. 22732443 10.1016/j.neuropharm.2012.06.027

[CIT0010] Duffy S Labrie V Roder JC (2008) D-serine augments NMDA-NR2B receptor-dependent hippocampal long-term depression and spatial reversal learning. Neuropsychopharmacology 33:1004–1018. 17625504 10.1038/sj.npp.1301486

[CIT0011] Ehlers CL Criado JR (2010) Adolescent ethanol exposure: does it produce long-lasting electrophysiological effects? Alcohol. 44:27–37. 20113872 10.1016/j.alcohol.2009.09.033 PMC2818286

[CIT0012] Fleming RL Li Q Risher ML Sexton HG Moore SD Wilson WA Acheson SK Swartzwelder HS (2013) Binge-pattern ethanol exposure during adolescence, but not adulthood, causes persistent changes in GABAA receptor-mediated tonic inhibition in dentate granule cells. Alcohol Clin Exp Res 37:1154–60. 23413887 10.1111/acer.12087 PMC3754782

[CIT0013] Ge Y Dong Z Bagot RC Howland JG Phillips AG Wong TP Wang YT (2010) Hippocampal long-term depression is required for the consolidation of spatial memory. Proc Natl Acad Sci U S A 107:16697–166702. 20823230 10.1073/pnas.1008200107 PMC2944752

[CIT0014] Griffiths S Scott H Glover C Bienemann A Ghorbel MT Uney J Brown MW Warburton EC Bashir ZI (2008) Expression of long-term depression underlies visual recognition memory. Neuron 58:186–194. 18439404 10.1016/j.neuron.2008.02.022

[CIT0015] Hingson RW Heeren T Winter MR (2006) Age at drinking onset and alcohol dependence: age at onset, duration, and severity. Arch Pediatr Adolesc Med 160:739–746. 16818840 10.1001/archpedi.160.7.739

[CIT0016] Izumi Y Kitabayashi R Funatsu M Izumi M Yuede C Hartman RE Wozniak DF Zorumski CF (2005a) A single day of ethanol exposure during development has persistent effects on bi-directional plasticity, N-methyl-D-aspartate receptor function and ethanol sensitivity. Neuroscience 136:269–279. 16181739 10.1016/j.neuroscience.2005.07.015

[CIT0017] Izumi Y Nagashima K Murayama K Zorumski CF (2005b) Acute effects of ethanol on hippocampal long-term potentiation and long-term depression are mediated by different mechanisms. Neuroscience 136:509–517. 16216426 10.1016/j.neuroscience.2005.08.002

[CIT0018] Karasawa J Hashimoto K Chaki S (2008) D-Serine and a glycine transporter inhibitor improve MK-801-induced cognitive deficits in a novel object recognition test in rats. Behav Brain Res 186:78–83. 17854919 10.1016/j.bbr.2007.07.033

[CIT0019] Kasanetz F Deroche-Gamonet V Berson N Balado E Lafourcade M Manzoni O Piazza PV (2010) Transition to addiction is associated with a persistent impairment in synaptic plasticity. Science 328:1709–1712. 20576893 10.1126/science.1187801

[CIT0020] Kemp A Manahan-Vaughan D (2004) Hippocampal long-term depression and long-term potentiation encode different aspects of novelty acquisition. Proc Natl Acad Sci U S A 101:8192–8197. 15150407 10.1073/pnas.0402650101 PMC419579

[CIT0021] Kemp N McQueen J Faulkes S Bashir ZI (2000) Different forms of LTD in the CA1 region of the hippocampus: role of age and stimulus protocol. Eur J Neurosci 12:360–366. 10651891 10.1046/j.1460-9568.2000.00903.x

[CIT0022] Kulkarny VV Wiest NE Marquez CP Nixon SC Valenzuela CF Perrone-Bizzozero NI (2011) Opposite effects of acute ethanol exposure on GAP-43 and BDNF expression in the hippocampus versus the cerebellum of juvenile rats. Alcohol 45: 461–471. 21367572 10.1016/j.alcohol.2010.12.004 PMC3119774

[CIT0023] Manahan-Vaughan D Braunewell KH (1999) Novelty acquisition is associated with induction of hippocampal long-term depression. Proc Natl Acad Sci U S A 96:8739–8744. 10411945 10.1073/pnas.96.15.8739 PMC17586

[CIT0024] Möykkynen T Korpi ER (2012) Acute effects of ethanol on glutamate receptors. Basic Clin Pharmacol Toxicol 111:4–13. 22429661 10.1111/j.1742-7843.2012.00879.x

[CIT0025] Liu L Wong TP Pozza MF Lingenhoehl K Wang Y Sheng M Auberson YP Wang YT (2004) Role of NMDA receptor subtypes in governing the direction of hippocampal synaptic plasticity. Science 304, 1021–1024. 15143284 10.1126/science.1096615

[CIT0026] Markwiese BJ Acheson SK Levin ED Wilson WA Swartzwelder HS (1998) Differential effects of ethanol on memory in adolescent and adult rats. Alcohol Clin Exp Res 22:416–421. 9581648

[CIT0027] Nelson TE Ur CL Gruol DL (2005) Chronic intermittent ethanol exposure enhances NMDA-receptor-mediated synaptic responses and NMDA receptor expression in hippocampal CA1 region. Brain Res 1048:69–79. 15919065 10.1016/j.brainres.2005.04.041

[CIT0028] Pascual M Blanco AM Cauli O Minarro J Guerri C (2007) Intermittent ethanol exposure induces inflammatory brain damage and causes long-term behavior alterations in adolescent rats. Eur J Neurosci 25:541–550. 17284196 10.1111/j.1460-9568.2006.05298.x

[CIT0029] Pascual M Boix J Felipo V Guerri C (2009) Repeated alcohol administration during adolescence causes changes in the mesolimbic dopaminergic and glutamatergic systems and promotes alcohol intake in the adult rat. J Neurochem 108:920–931. 19077056 10.1111/j.1471-4159.2008.05835.x

[CIT0030] Roberto M Nelson TE Ur CL Gruol DL (2002) Long-term potentiation in the rat hippocampus is reversibly depressed by chronic intermittent ethanol exposure. J Neurophysiol 87:2385–2397. 11976376 10.1152/jn.2002.87.5.2385

[CIT0031] Roberto M Nelson TE Ur CL Brunelli M Sanna PP Gruol DL (2003) The transient depression of hippocampal CA1 LTP induced by chronic intermittent ethanol exposure is associated with an inhibition of the MAP kinase pathway. Eur J Neurosci 17:1646–54. 12752382 10.1046/j.1460-9568.2003.02614.x

[CIT0032] Schneider M (2013) Adolescence as a vulnerable period to alter rodent behavior. Cell Tissue Res. 354:99–106. 23430475 10.1007/s00441-013-1581-2

[CIT0033] Shipton OA Paulsen O (2013) GluN2A and GluN2B subunit-containing NMDA receptors in hippocampal plasticity. Philos Trans R Soc Lond B Biol Sci 369: 20130163. 24298164 10.1098/rstb.2013.0163 PMC3843894

[CIT0034] Silveri MM Spear LP (1998) Decreased sensitivity to the hypnotic effects of ethanol early in ontogeny. Alcohol Clin Exp Res 22:670–676. 9622449 10.1111/j.1530-0277.1998.tb04310.x

[CIT0035] Taukulis HK Bigney EE Fry MD Hooper C (2014) Object memory impairment at post-drug Day 15 but not at Day 1 after a regimen of repeated treatment with oral methylphenidate. Neurosci Lett 566C:252–256. 24631430 10.1016/j.neulet.2014.03.001

[CIT0036] Thinschmidt JS Walker DW King MA. (2003) Chronic ethanol treatment reduces the magnitude of hippocampal LTD in the adult rat. Synapse 48:189–197. 10.1002/syn.10203 12687638

[CIT0037] Townshend JM Duka T (2005) Binge drinking, cognitive performance and mood in a population of young social drinkers. Alcohol Clin Exp Res 29:317–325. 15770105 10.1097/01.alc.0000156453.05028.f5

[CIT0038] Watanabe M Yoshikawa M Takeyama K Hashimoto A Kobayashi H Suzuki T (2010) Subchronic administration of ketamine decreases the mRNA expression of serine racemase in rat brain Tokai J Exp Clin Med 35:137–143. 21319044

[CIT0039] White AM Swartzwelder HS (2005) Age-related effects of alcohol on memory and memory-related brain function in adolescents and adults. Recent Dev Alcohol 17:161–76. 15789865 10.1007/0-306-48626-1_8

[CIT0040] Wozniak DF Hartman RE Boyle MP Vogt SK Brooks AR Tenkova T Young C Olney JW Muglia LJ (2004) Apoptotic neurodegeneration induced by ethanol in neonatal mice is associated with profound learning/memory deficits in juveniles followed by progressive functional recovery in adults. Neurobiol Dis 17:403–414. 15571976 10.1016/j.nbd.2004.08.006

[CIT0041] Zhang Z Gong N Wang W Xu L Xu TL (2008) Bell-shaped D-serine actions on hippocampal long-term depression and spatial memory retrieval. Cereb Cortex 18:2391–2401. 18281302 10.1093/cercor/bhn008

